# Effects of a loaded change of direction training program on physical performance in U-19 elite soccer players

**DOI:** 10.1371/journal.pone.0335148

**Published:** 2025-10-17

**Authors:** Víctor Martín, Mehdi Ben Brahim, Ariadna Hernaiz-Sánchez, Hussain Yassin, Alejandro Sal-de-Rellán

**Affiliations:** 1 Faculty of Health Sciences, Universidad Isabel I, Burgos, Spain; 2 Health and Physical Education Department, Prince Sultan University, Riyadh, Kingdom of Saudi Arabia; 3 Department of Education and Educational Innovation, Faculty of Law, Education and Humanities, Universidad Europea de Madrid, Madrid, Spain; Portugal Football School, Portuguese Football Federation, PORTUGAL

## Abstract

Changes of direction are one of the most repeated actions during football matches, which is why the inclusion of these actions in the training of the football player must be a fundamental aspect. The aim of this study was to assess the effects of 6-week a loaded change of direction (COD) movements training on speed, jump, COD speed, and repeated sprint ability (RSA) in soccer players. Twenty-eight male soccer players (age: 19.12 ± 0.75 years; height: 1.75 ± 0.06 m; body mass: 72.78 ± 4.87 kg; systematic practice: 8.62 ± 1.33 years) were randomly assigned to an experimental group (EG, n = 14) or a control group (CG, n = 14). The research was conducted during a training camp. During the intervention period, the EG performed two weekly sessions of loaded change of direction training, while the CG performed FIFA 11 prevention program. EG significantly improved the performance of 5-m, CMJ, SJ, Illinois and the percentage of decrement (%Dec and %Dec-COD). However, CG only showed significant improvements in CMJ and SJ. Between-groups analysis revealed differences in favor of the EG in 5-m, CMJ, SJ. The main results show that the effect of loaded COD movements training using a weighted vest on the physical performance of soccer players is significantly greater compared to the FIFA 11 prevention program. This study shows that including an additional load in COD exercises is a good method to increase performance on key variables for soccer players.

## Introduction

Soccer is characterized by short sprints and frequent change of direction (COD) movements [1,2]. According to the scientific literature, soccer players perform 95% of sprints in less than 10-m and 76% in less than 5-m [3]. In addition, an average of 726 changes of direction with different angles and in various directions are performed during the match [1]. As we can observe at a quantitative level, the COD is a very important aspect in the performance of the soccer player since there is a high demand in competition. Furthermore, it has been shown that linear actions followed by deceleration and turn (i.e., COD) are the most common movements preceding goals [4].

According to the Chaabene et al. [5] model, COD speed is affected by several factors, such as technique, straight sprinting speed, leg muscle qualities (i.e., reactive strength, concentric strength and power, eccentric strength, and left-right muscle imbalance), and anthropometry. In order to improve COD skills, many researchers have come up with training programs that focus on developing these factors [6]. These programs have been tested and found to be very reliable [7,8]. For instance, young soccer players were assessed after a 12-week conditioning program involving speed, agility, and quickness training. After this, the COD improved, while in the control group, which performed the usual soccer training, no improvements were observed [9]. In another study, COD speed improved significantly in soccer players after 6-week COD movements training [10]. Although the means of training used in these investigations may be beneficial, implementing a training strategy that adheres to the principle of specificity (i.e., a conditioning program that emphasizes similar motor patterns used during the game) can lead to a greater positive transfer of training to athletic performance. In this regard, resisted sprint training, which involves soccer players sprinting with added load without substantial changes in running technique [11], has been used to improve sprint, COD, repeated sprint ability (RSA), jump, or endurance [12,13].

To the best of our knowledge, few studies have used this type of training in exercises with a change of direction. Recently, a study performed by Rodríguez-Osorio et al. [14] compared the effects of resisted change of direction movements using different relative loads (i.e., without external load, with 12.5% and 50% body mass) on soccer players. After the post-training evaluation, the authors observed that the training group with a moderate load (i.e., 12.5% body mass) had the most positive effects on COD skills, sprinting performance, and jumping ability. However, they only used one exercise (V-cut test) as a means of training, always using the same angles (45º) in the COD. Therefore, with the idea of including different angles in loaded COD training and making a more complete training, the aim of this study was to evaluate the effects of a 6-week training program with loaded COD movements on different angles on soccer players’ physical performance. Based on this, we hypothesized that the use of loaded COD exercises (i.e., with a weighted vest) over different angles would provide greater training variability, thus improving the performance of our players to a greater extent than an injury prevention training (i.e., FIFA 11 prevention program).

## Materials and methods

### Participants

Twenty-eight male soccer players (age: 19.12 ± 0.75 years; height: 1.75 ± 0.06 m; body mass: 72.78 ± 4.87 kg; systematic practice: 8.62 ± 1.33 years) from the Tunisian national U-19 team volunteered to participate in this study. Using a controlled and randomized study design, players were assigned in experimental group (EG, n = 14) or control group (CG, n = 14; https://www.randomizer.org). Inclusion criteria were no injuries in the past six months that limited sports participation for over seven days and to have participated in 90% of the training sessions. Before signing the consent form for testing, participants were informed of the experimental procedures, risks, and benefits associated with the investigation. The university’s ethics committee (Ui1-PI104) approved the study and performed it in accordance with the Declaration of Helsinki (2013).

### Design

The investigation was conducted during a training camp. The training program was conducted for 6-weeks with a frequency of twice a week (from 22/07/2024–15/09/2024). During the week preceding the experiment, players became familiarized with the testing procedures. At the beginning and the end of this intervention, 5 tests were performed: a linear sprint test, vertical jump tests, a COD speed test, a repeated sprint ability test (RSA test), and a repeated shuttle sprint ability test (RSA-COD test). The tests were performed in three nonconsecutive days (i.e., 48–72 h between testing day). The first day, players performed the vertical jump tests and RSA test, the second day, they performed linear sprint test and COD speed test, and the third day they performed RSA-COD test. All testing sessions were conducted outdoors, on natural grass, at the same habitual training time (i.e., 10:00–12:00 AM) and under similar environmental conditions (23–25 ºC), with the same sports clothes, and by the same testers. Before testing, a general and specific warm-up routine was performed, involving 3-min of jogging, followed by 5-min of dynamic and ballistic stretching, and 7-min of progressive sprints and accelerations [15].

During the experimental period, both groups (EG and CG) followed a weekly standard training program. A common microcycle consists of a recovery, strength, endurance, speed and activation sessions (Table 1). Additionally, there was one soccer match played at the weekend. These training sessions were conducted with the same coach. In order to minimize the impact of uncontrollable factors, players were instructed to maintain their usual lifestyle and regular food habits prior to and during the duration of the research.

**Table 1:** Typical weekly training program during the 6 weeks of intervention.

Monday	Tuesday	Wednesday	Thursday	Friday	Saturday	Sunday
Recovery (75 min)	Rest	Strength (80 min)	Endurance (90 min)	Speed (70 min)	Activation (60 min)	Match
Warm-up: 15 min		Warm-up: 15 min	Warm-up: 15 min	Warm-up: 15 min	Warm-up: 15 min	
60 min Starters		15 min	Positional games: 20 min	15 min	Activation exercises: 10 min	
Aerobic endurance: 15 min		EG: Loaded change of direction training.	Large-sided game: 20 min	EG: Loaded change of direction training.	Tactical drills: 20 min	
Injury prevention: 30 min		CG: FIFA 11 prevention.	Simulated game: 35 min	CG: FIFA 11 prevention.	Strategy: 15 min	
Stretching 15 min		Positional games: 20 min		Medium-sided game: 20 min		
Nonstarters		Small-sided games: 30 min		Tactical drills: 20 min		
Injury prevention: 15 min						
Small-sided games: 30 min						
Speed endurance: 15 min						

#### Training intervention.

Only EG performed a twice-weekly session of loaded change of direction training, while CG performed FIFA 11 prevention program (i.e., core stability, balance, plyometrics and strength) [16]. The EG training consisted of three exercises. One set of each exercise, with a weighted vest (12.5% body mass) [14], was performed (Table 2). Although each exercise covered a different distance and involved different angles, all of them had four changes of direction (CODs). Strong verbal encouragement was provided to each player in all sets. The training volume progressed from the first to the fourth week (i.e., from 12 CODs to 36 CODs) and was reduced in the last two weeks to achieve a tapering effect.

**Table 2:** Summary of training load in the intervention period by EG.

Weeks	Sets	Rep.	Total distance per training (m)	Nº CODs per training	Recovery between repetitions (sec)	Recovery between sets (min)	Exercises
1	3	1	90	12	–	3	Exercise 1 was performed on 1^st^ set. Exercise 2 was performed on 2^nd^ set. Exercise 3 was performed on 3^rd^ set.
2	3	2	180	24	30	3	
3	3	2	180	24	30	3	
4	3	3	270	36	30	3	
5	3	2	180	24	30	3	
6	3	1	90	12	–	3	

#### Exercise nº1.

**Fig 1:**
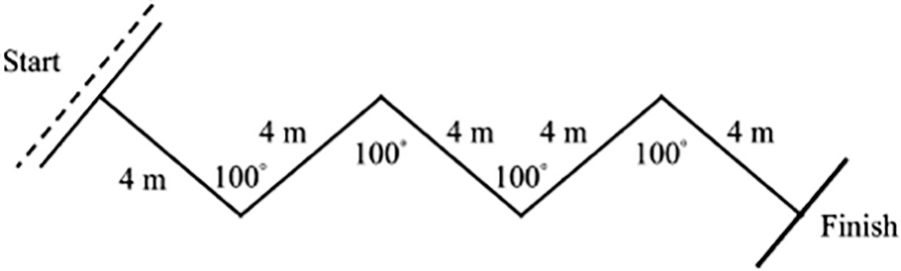
Exercise nº1 was performed by the EG during the intervention period.

Exercise nº1 consisted of four 100º CODs at every 4-m as shown in Fig 1. The total distance covered was 20-m each repetition [17].

#### Exercise nº2.

**Fig 2:**
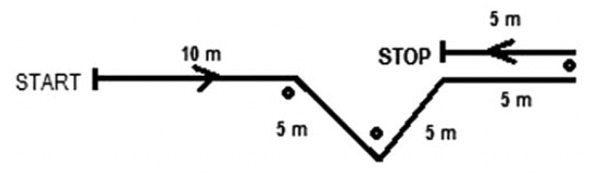
Exercise nº2 was performed by the EG during the intervention period.

As seen in Fig 2, exercise nº2 required subjects to sprint 30-m with four CODs during the run [18].

#### Exercise nº3.

**Fig 3:**
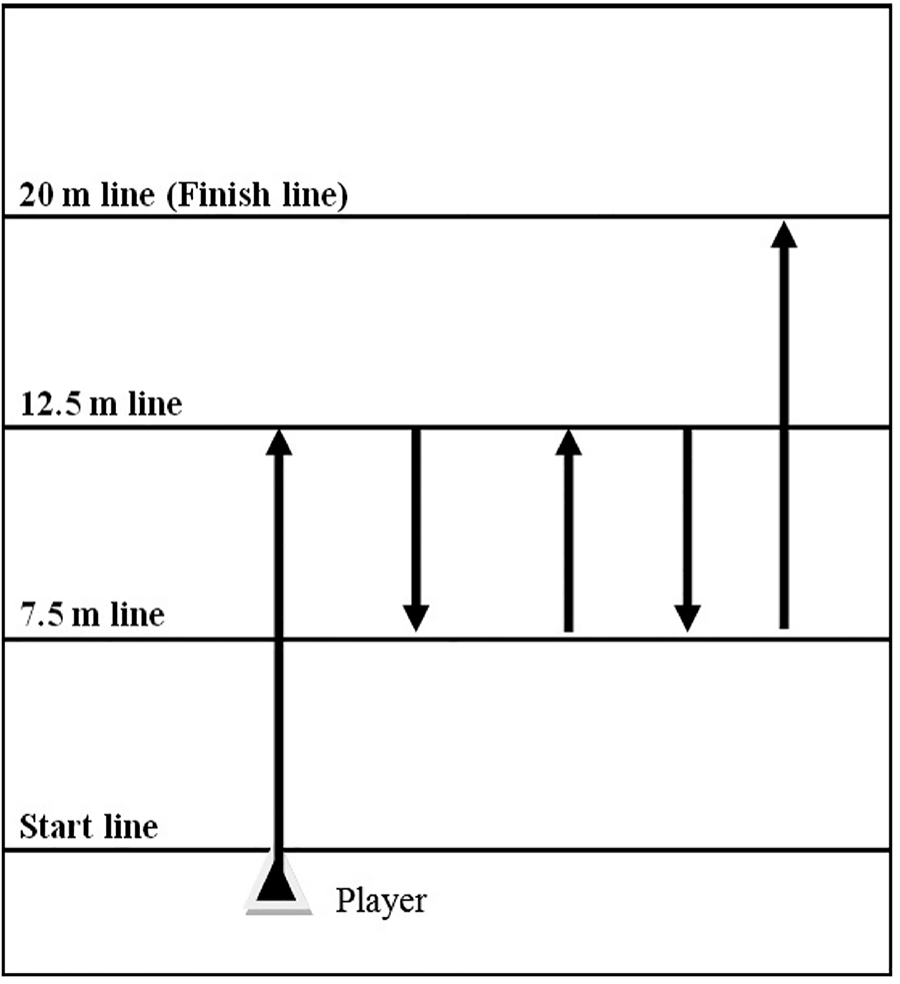
Exercise nº3 was performed by the EG during the intervention period.

Exercise nº3 involved a total running distance of 40-m and included four 180º turns (Fig 3), with the players sprinting from 0–12.5 m, back to the 7.5-m line, forwards to the 12.5-m line, back to the 7.5-m line for the last time and finally forwards to the finish line at 20-m [19].

### Physical fitness tests

#### Linear sprint test.

Players started from a standing position, 0.5-m behind the first timing gate (WittySEM, Microgate®, Bolzano, Italy), before running at maximal speed to the second timing gate. Participants were encouraged to execute two maximum sprints of 30-m, with 5-m and 10-m split times, being allowed a 2-min of passive recovery between trials. The fastest time was retained for analysis.

#### COD speed test.

Illinois test was used to evaluate the pre-planned COD movements the athletes performed as it has been described in detail [20]. Times were recorded with timing gates (WittySEM, Microgate®, Bolzano, Italy) at the start and the end of test. The faster of two successful trials was employed for further analysis. Between each trial there was a 2-min of passive recovery.

#### Vertical jump tests.

Jumping height was calculated through flight time (Optojump, Microgate, Bolzano, Italy) in SJ and CMJ. Players began the SJ with a knee angle of 90º, avoiding downward movement. Subsequently, they performed a vertical jump by driving themselves up, keeping straight legs throughout the flight time. CMJ was similar but began from upright, followed by a downward movement to a knee angle of 90º then a maximal upwards jump [21]. Each player performed two jumps with two minutes of passive recovery between jumps of each test. The best jump from the SJ and CMJ was selected for subsequent analysis.

#### Repeated sprint ability.

The protocol was previously used by Fernandes-da-Silva et al. [22]. It consisted of seven repeated 34.2-m sprints separated by 25 s of active recovery (i.e., jogging back to the starting line within approximately 20 s, to allow 4–5 s of passive recovery before the commencement of the next sprint repetition). Players used a standing start 0.5 m behind the timing lights (WittySEM, Microgate®, Bolzano, Italy). Players were given verbal encouragement to run as fast as possible for each of the eight sprints and constant verbal feedback was provided during the recovery run. The best (RSA_b_), the mean (RSA_m_) and the percentage of decrement (%Dec) in sprint time were retained for subsequent analysis. %Dec was calculated using the following formula: [100 x (total sprint time/ideal sprint time)] – 100, where total sprint time is a sum of sprint times from all sprints and ideal sprint time is the number of sprints • fastest sprint time [23].

#### Repeated shuttle sprint ability test.

The RSA-COD test comprised 6 bouts of 2 x 20-m shuttle sprints (i.e., with 180º COD) and 20 seconds passive rest between bouts [24]. Each sprint was started by the participants from a standing start, with their front foot planted 0.5-m behind the timing gate (WittySEM, Microgate®, Bolzano, Italy). The best (RSA-COD_b_), the mean (RSA-COD_m_) and the percentage of decrement (%Dec-COD) in sprint time were retained for subsequent analysis. %Dec-COD was calculated using the same formula than %Dec [23].

### Statistical analysis

Descriptive analysis was presented as mean ± standard deviation (SD). For the assumption of normality, the Shapiro-Wilk test was used, which confirmed that the data had a normal distribution, while the Levene test showed that the variance was homogeneous. All variables were normally distributed. A Paired-samples *t*-test was used to evaluate within-group differences, and an analysis of covariance (ANCOVA) was performed to detect possible between-group differences (i.e., EG vs CG), assuming baseline values as covariates. Effect sizes (ES) were calculated using Cohen’s ES and were interpreted as follows: < 0.2, trivial; 0.20 to 0.49, small; 0.50 to 0.80, moderate and >0.80, large [25]. Statistical significance was set at *p* < 0.05. Statistical analyses were performed by JASP software version 0.10.2 (Amsterdam, Netherlands) for Macintosh.

## Results

Changes in assessment tests before (baseline) and after (post-training) the 6-week intervention period in both groups are shown in Table 3.

**Table 3:** Assessment test before (baseline) and after (post-training) the 6-week intervention period in both groups.

EG						CG					Between group differences	
Variable	Baseline mean ± SD	Post training mean ± SD	Δ Mean ± SD (95% CI)	*p*	ES	Baseline mean ± SD	Post training mean ± SD	Δ Mean ± SD (95% CI)	*p*	ES	F	*p*
5-m (s)	1.085 ± 0.082	1.047 ± 0.052	0.038 ± 0.014	0.016	0.742	1.096 ± 0.066	1.066 ± 0.068	0.029 ± 0.025	0.269	0.309	4.209	0.049
10-m (s)	1.886 ± 0.093	1.871 ± 0.093	0.015 ± 0.032	0.655	0.122	1.889 ± 0.119	1.889 ± 0.095	0.000 ± 0.049	1.000	0.000	1.013	0.324
30-m (s)	4.353 ± 0.289	4.264 ± 0.189	0.088 ± 0.112	0.441	0.212	4.361 ± 0.315	4.325 ± 0.215	0.036 ± 0.097	0.719	0.098	0.734	0.400
SJ (cm)	29.243 ± 4.782	31.370 ± 4.536	− 2.127 ± 0.387	< .001	− 1.470	28.371 ± 3.684	29.871 ± 3.640	− 1.500 ± 0.184	< .001	−2.186	330.4	< .001
CMJ (cm)	33.143 ± 4.782	37.357 ± 4.536	− 4.214 ± 0.536	< .001	− 2.010	32.157 ± 4.161	34.314 ± 3.718	− 2.157 ± 0.386	< .001	−1.493	132.6	< .001
Illinois (s)	16.896 ± 1.038	15.974 ± 0.182	0.922 ± 0.295	0.008	0.835	16.879 ± 1.019	16.529 ± 0.794	0.349 ± 0.364	0.355	0.257	0.397	0.534
RSA_b_ (s)	5.955 ± 0.225	5.972 ± 0.273	− 0.017 ± 0.074	0.822	−0.061	6.034 ± 0.206	6.172 ± 0.239	− 0.138 ± 0.073	0.081	−0.506	2.94	0.099
RSA_m_ (s)	6.401 ± 0.273	6.277 ± 0.300	0.123 ± 0.084	0.165	0.393	6.543 ± 0.238	6.611 ± 0.249	− 0.068 ± 0.078	0.399	−0.233	3.66	0.067
%Dec	7.487 ± 2.171	5.111 ± 1.945	2.379 ± 0.684	0.004	0.928	8.444 ± 2.418	7.122 ± 1.863	1.321 ± 0.809	0.127	0.436	0.360	0.554
RSA-COD_b_ (s)	7.975 ± 0.225	7.952 ± 0.224	0.023 ± 0.098	0.820	0.062	7.976 ± 0.198	8.086 ± 0.315	− 0.110 ± 0.105	0.312	−0.281	1.20	0.284
RSA-COD_m_ (s)	8.592 ± 0.238	8.422 ± 0.237	0.170 ± 0.090	0.081	0.505	8.599 ± 0.208	8.641 ± 0.271	− 0.041 ± 0.077	0.605	−0.142	0.582	0.453
%Dec-COD	7.745 ± 1.571	5.925 ± 1.904	1.819 ± 0.770	0.035	0.631	7.825 ± 2.058	6.901 ± 2.067	0.926 ± 0.613	0.155	0.403	0.125	0.727

EG = Experimental Group; CG = control group; SD = standard deviation; Δ: real change between pre- and post-training performance; CI = confidence interval; *p* = level of significance; ES = effect size; 5-m = 5 meters sprint test; 10-m = 10 meters sprint test; 30-m = 30 meters sprint test; SJ = squat jump; CMJ = countermovement jump; Illinois = Illinois Agility Test; RSA_b_, RSA_m_, and %Dec = best, mean, and percentage of decrement in the repeated sprint ability test; RSA-COD_b_, RSA-COD_m_, and %Dec-COD = best, mean, and percentage of decrement in the repeated shuttle sprint ability test.

### Linear sprint test

In the sprinting ability, GE had significant improvements in 5-m (p = 0.016; ES = 0.742, moderate), with no significant improvements in the other two distances (10- and 30-m). For the CG, no significant improvements were observed in any distance of the linear sprint (i.e., 5-m, 10-m and 30-m). Between-groups analysis revealed significant differences in the sprint test in 5-m (F = 4.209; p = 0.049) in favor of EG.

### Jumping test

In the jumping test, both groups (EG and CG) significantly improved in SJ (EG: *p* < .001; ES = −1.470, large; CG: *p* < .001; ES = −2.186, large) and CMJ (EG: *p* < .001; ES = −2.010, large; CG: *p* < .001; ES = −1.493, large). Between-groups analysis revealed significant differences in the jumping test in SJ (F = 330.4; *p* < .001) and CMJ (F = 132.6; *p* < .001) in favor of EG.

### COD speed test

In the Illinois test, the EG significantly improved performance in the ability to change direction (*p* = 0.08; ES = 0.835, large). However, no significant improvements were found either in CG or between groups.

### RSA tests

In the RSA tests, EG significantly improved performance in both percentage of decrement (%Dec: **p* *= 0.004; ES = 0.928, large; %Dec-COD: **p* *= 0.035; ES = 0.631, moderate). However, CG did not significantly improve performance on the RSA variables. Between-group analysis revealed no significant differences.

## Discussion

This study investigated the effects of 6-week loaded COD movements training on soccer players’ physical performance. The main findings of this study indicate that EG improved in acceleration ability (5-m), jumping ability (SJ and CMJ), COD speed (Illinois), and percentage of decrement (%Dec and %Dec-COD) in RSA tests. Moreover, CG enhanced jumping ability (SJ and CMJ). Regarding the analysis between groups, significant differences were found in 5-m, SJ and CMJ in favor of EG.

Regarding the findings from the sprint-based testing, the present study only demonstrated improvements in 5-m performance in intragroup analysis (i.e., EG) and intergroup analysis (i.e., EG vs. CG). This finding was anticipated given that significant improvements in sprint performance (5-m and 20-m) were observed as a result of a resisted sprints intervention with youth soccer players [26]. However, our study did not improve 10-m and 30-m sprint performance. It is possible that our training, based on COD movements where the maximum distance covered in a straight line was 12.5-m, will not cause the adaptations necessary to improve speed over a distance greater than 5-m. Additionally, it has been believed that this type of training load (between 5 and 12.5% of body mass) improves performance in the maximum speed phases (20- and 30-m), but we must keep in mind that in our study, the forces generated during the change of direction actions (i.e., accelerate, decelerate, and re-accelerate) implement the weighted vest load. Therefore, the overload of our training (i.e., the weighted vest plus the forces created by changing directions) may be the cause of the induced improvements in 5-m. This is because high training loads (> 20% body mass) in resisted sprint exercises are mostly meant to improve the acceleration phases of sprinting [27,28].

Despite not aiming to improve jumping ability, both groups improved their CMJ and SJ performance. The program used by the CG incorporated a plyometric stimulus into its training content. It is possible that including this content in the CG intervention may have led to the improvements obtained in jumping ability [29]. In addition, as in our study, the use of weighted vest training has already led to improvements in jumping performance in soccer players [14,30]. It has been justified that the use of the weighted vest in activities such as resisted sprints have a greater vertical application of force compared to the use of sleds [31], which benefits the direction of the forces used in a vertical jump (i.e., CMJ and SJ). In addition, the EG seriously involved the knee extensor musculature in all the exercises conducted during the intervention period. These mechanical and muscular implications have traditionally been related to the improvement of lower body power, such as that developed in the jumping ability measured in this research [32,33].

Because specificity is a crucial component of training, the exercises performed in soccer must simulate the biomechanical and physiological demands of the sport [34,35]. During a game, soccer players engage in a variety of COD, sprints, accelerations, and decelerations [2,36]. Therefore, as our results show, the application of this type of training (i.e., loaded COD movements) that meets the specificity requirements (i.e., similar motor patterns used during the game) obtains improvements in the ability to rapidly change direction. These findings are in line with those of other investigations. For example, young soccer players improved COD speed after six weeks of COD exercise training [10]. In this line, but with professional soccer players, an elastic cords resistance system (Vertimax ®) was included in this type of training [13]. These authors found that both resisted and unresisted training, improved the performance in the COD. It is very likely that the tension generated by elastic cords may not be held constant, producing an insufficient training load for the resisted training group, which was not able to perform better. However, in our study, the use of a weighted vest may have increased the overload during the training program by inducing greater production of deceleration and re-acceleration forces, leading to an improvement in COD performance [37]. In addition, greater eccentric strength in the knee extensors has been shown to improve the ability to decelerate, which greatly contributes to increased COD performance [5,38].

As a result of an increase in high-intensity actions in soccer [39], RSA has been recognized as one of the most important physical abilities [40]. The results of our study show a significant improvement in fatigue indices (%Dec and %Dec-COD) for EG. In addition, the EG improves performance in the mean time of the RSA tests. These results are in line with those found by other research conducted with resisted sprint training [12] or sprint-based training with COD [41]. The training made, with very high intensity actions and short recovery periods between repetitions (30 s), favours adaptations in anaerobic metabolism, this being a determining factor in the performance of the RSA [42]. Although we must be cautious when interpreting the results, as different ways of calculating the fatigue index have been used, which causes great controversy in the scientific literature [43]. Despite this, it seems clear that a greater capacity for recovery between sprints produces a better performance of soccer players in competition [44].

There are certain limitations that should be mentioned in this study: (1) We didn’t use an unloaded COD movements training group to compare the effect in relation to EG and CG; (2) although the number of participants in our study was similar to other investigations that have evaluated different training methods in team sports, our sample size was somewhat limited. Therefore, future studies are necessary to gather more evidence on the adaptations resulting from training loaded COD movements, given the scarcity of research on the incorporation of weighted vests in COD-based exercises.

### Practical application

Within a soccer-training, there is a restricted amount of time allocated for strength and conditioning exercises. It is essential to have tactics that save time and improve multiple specific actions simultaneously. Therefore, we suggest that including an additional load (i.e., a weighted vest) in COD exercises is a good method to increase performance in key variables for soccer players (i.e., acceleration, change of direction, jumping, or the ability to repeat sprints). Finally, strength and conditioning coaches could include this training twice a week before technical-tactical soccer training.

### Conclusions

The present study revealed that the effect of loaded COD movements training using a weighted vest on the physical performance of soccer players is significantly greater compared to the FIFA 11 prevention program. In conclusion, technical-tactical soccer training should be complemented, in addition to training aimed at injury prevention, by specific conditional training that aims to optimize variables related to soccer performance.

## Supporting information

S1 Data Data availability document. (CSV)
